# A gradient of nutrient enrichment reveals nonlinear impacts of fertilization on Arctic plant diversity and ecosystem function

**DOI:** 10.1002/ece3.2863

**Published:** 2017-03-22

**Authors:** Case M. Prager, Shahid Naeem, Natalie T. Boelman, Jan U. H. Eitel, Heather E. Greaves, Mary A. Heskel, Troy S. Magney, Duncan N.L. Menge, Lee A. Vierling, Kevin L. Griffin

**Affiliations:** ^1^ Department of Ecology, Evolution, and Environmental BiologyColumbia UniversityNew YorkNYUSA; ^2^ Department of Earth and Environmental SciencesColumbia UniversityNew YorkNYUSA; ^3^ Lamont‐Doherty Earth ObservatoryColumbia UniversityPalisadesNYUSA; ^4^ Geospatial Laboratory for Environmental DynamicsDepartment of Natural Resources and SocietyUniversity of IdahoMoscowIDUSA; ^5^ McCall Outdoor Science SchoolUniversity of IdahoMcCallIDUSA; ^6^ Ecosystems CenterMarine Biological LaboratoryWoods HoleMAUSA; ^7^ Jet Propulsion LaboratoryCalifornia Institute of TechnologyPasadenaCAUSA

**Keywords:** Arctic, climate change, ecosystem function, ecosystem respiration, gross primary productivity, net ecosystem CO_2_ exchange, plant diversity

## Abstract

Rapid environmental change at high latitudes is predicted to greatly alter the diversity, structure, and function of plant communities, resulting in changes in the pools and fluxes of nutrients. In Arctic tundra, increased nitrogen (N) and phosphorus (P) availability accompanying warming is known to impact plant diversity and ecosystem function; however, to date, most studies examining Arctic nutrient enrichment focus on the impact of relatively large (>25x estimated naturally occurring N enrichment) doses of nutrients on plant community composition and net primary productivity. To understand the impacts of Arctic nutrient enrichment, we examined plant community composition and the capacity for ecosystem function (net ecosystem exchange, ecosystem respiration, and gross primary production) across a gradient of experimental N and P addition expected to more closely approximate warming‐induced fertilization. In addition, we compared our measured ecosystem CO_2_ flux data to a widely used Arctic ecosystem exchange model to investigate the ability to predict the capacity for CO_2_ exchange with nutrient addition. We observed declines in abundance‐weighted plant diversity at low levels of nutrient enrichment, but species richness and the capacity for ecosystem carbon uptake did not change until the highest level of fertilization. When we compared our measured data to the model, we found that the model explained roughly 30%–50% of the variance in the observed data, depending on the flux variable, and the relationship weakened at high levels of enrichment. Our results suggest that while a relatively small amount of nutrient enrichment impacts plant diversity, only relatively large levels of fertilization—over an order of magnitude or more than warming‐induced rates—significantly alter the capacity for tundra CO_2_ exchange. Overall, our findings highlight the value of measuring and modeling the impacts of a nutrient enrichment gradient, as warming‐related nutrient availability may impact ecosystems differently than single‐level fertilization experiments.

## 1 Introduction

High‐latitude ecosystems have experienced rapid warming in recent decades. Mean Arctic surface temperature has increased by 2°C over the past 50 years compared to an increase of approximately 0.72°C in global mean surface temperature (IPCC 2013). Arctic tundra is warming rapidly due to a network of positive feedbacks among regional temperature, water vapor, albedo, and associated variations in snow depth, permafrost thaw, and sea ice extent (Chapin et al., 2005; Hinzman et al., 2013; Serreze & Francis, 2006). Consequently, Arctic tundra ecosystems are predicted to be affected more by warming than any other terrestrial ecosystem (IPCC 2013).

The effects of Arctic warming are complex and diverse, including a deepening active layer, increased soil nutrient mineralization and subsequent fertilization of a historically nitrogen (N)‐ and phosphorus (P)‐limited landscape (Chapin, 1991; Shaver & Chapin, 1986). Greater nutrient availability is thought to lead to shifts in plant community composition and physical structure due to increases in the relative abundance of woody, deciduous shrub species, with consequences for key ecosystem functions such as carbon (C) and nutrient cycling (Hobbie & Chapin, 1998; Myers‐Smith et al., 2011; Rastetter et al., 1991). In addition, increased nutrient availability is expected to stimulate primary production, enhancing aboveground biomass and ecosystem C gain and belowground productivity and C cycling (Hill & Henry, 2011; Hobbie, Nadelhoffer, & Hogberg, 2002), as has been shown by modeling efforts (Jiang et al., 2016). However, recent assessments suggest that, regardless of shifts in aboveground biomass and ecosystem productivity, concurrent increases in organic matter decomposition are weakening the strength of the Arctic CO_2_ sink (Hayes et al., 2011), and the region is likely to become a net C source to the atmosphere by 2100 (Abbott et al., 2016). As high‐latitude ecosystems contain twice as much C as there is presently in the atmosphere (Tarnocai et al., 2009; Zimov, Schuur, & Chapin, 2006), more than three times the C in global forest biomass (Houghton, 2007), and between a quarter and a third of the globe's total C pools (Carvalhais et al., 2014; Schimel et al., 2015), understanding the ecological consequences of rapid warming and a growing nutrient pool for leaf, community, and ecosystem processes across Arctic tundra ecosystems is paramount.

The majority of nutrient addition experiments—across all ecosystems—aim to examine the extent of nutrient limitation on annual net primary productivity (NPP) (LeBauer & Treseder, 2008). To do so, nutrients are often added at levels that far exceed plant demand—at times an order of magnitude greater than predicted deposition or warming‐induced increases in nutrient availability (Hobbie et al., 2002). Experimental N and P additions have been used to simulate enrichment in Arctic tundra ecosystems as warming is thought to increase nutrient availability via increases in active layer depths and accelerations in the decomposition of soil organic matter (Aerts, Cornelissen, & Dorrepaal, 2006; Hartley, Neill, Melillo, Crabtree, & Bowles, 1999; Schimel, Bilbrough, & Welker, 2004). Such large dose, long‐term fertilization experiments (i.e., annual additions of ≥10 g m^−2^ year^−1^ N and ≥5 g m^−2^ year^−1^ P) across varying Arctic tundra types have documented increases in NPP and pronounced shifts in plant community composition and physical structure over time (Boelman, Stieglitz, Griffin, & Shaver, 2005; Boelman et al., 2003; Shaver & Chapin, 1986; Shaver et al., 1998) often occurring in connection with increases in the abundance of deciduous woody shrub species and decreases in evergreen, grass/sedge, and moss cover (Shaver & Chapin, 1986; Shaver et al., 1998).

Shifts in the evenness and dominance of plant species, and declines in plant diversity, are often attributable to shifts in competitive interactions between plant species with increasing nutrient availability (Tilman, 1984, 1987) and the competitive displacement of low stature species due to light limitation (Goldberg & Miller, 1990). One study has shown that high levels of N and P fertilization doubled NPP, but soil C—a much larger pool—decreased substantially, resulting in a net decrease of ecosystem C storage (Mack, Schuur, Bret‐Harte, Shaver, & Chapin, 2004). In contrast, examination of more gradual shifts in nutrient availability via long‐term warming showed increases in plant biomass and dominance of woody shrub species with no changes in total soil C and N pools, ultimately increasing net ecosystem C storage after 20 years (Sistla et al., 2013). However, it is unclear how much of this response was driven by direct effects of temperature increases versus indirect effects of warming‐related nutrient enrichment (Sistla et al., 2013). In addition, large‐scale experimental and observational warming studies have documented increases in deciduous shrub cover that is often indirectly attributed to nutrient enrichment (Elmendorf, Henry, Hollister, Bjork, Bjorkman, et al. 2012; Elmendorf, Henry, Hollister, Bjork, Boulanger‐Lapointe, et al. 2012). While substantial variation in the structure and composition of tundra vegetation exists, previous work has illuminated relatively consistent relationships between productivity and biomass, and canopy leaf area and nutrient use or allocation (Shaver & Chapin, 1995; Shaver et al., 1998; Williams & Rastetter, 1999). These findings point to the functional convergence of canopies—suggesting similar controls over canopy‐level C exchange regardless of any compositional differences in plant communities (Shaver, Street, Rastetter, Van Wijk, & Williams, 2007; Street, Shaver, Williams, & Van Wijk, 2007; Williams & Rastetter, 1999; Williams et al., 2001)—regardless of the impacts of any variation in resource availability not captured by canopy leaf area.

Monitoring plant community and ecosystem responses across a gradient of fertilization may reveal important dynamics and relationships between plant nutrient availability and use. For example, there may be a point at which plant nutrient availability or uptake outpaces utilization, or nonlinear relationships may emerge between nutrient enrichment and ecosystem function (Aber et al., 1998; Bai et al., 2010). Further, experiments that have added a range of N and P levels report shifts in diversity or biomass at all levels of nutrient addition (Bowman, Gartner, Holland, & Wiedermann, 2006; Britton & Fisher, 2007; Tilman, 1987), suggesting that ecosystem properties or processes may be impacted by low levels of enrichment (Clark & Tilman, 2008). Addressing both the magnitude and variability of nutrient enrichment in a changing world is important if we are to improve our overall understanding of the effects of nutrient availability on plant communities and ecosystem function.

The few incremental nutrient addition experiments that have been conducted in Arctic tundra have found community‐level responses to small differences in nutrient enrichment. One study in Northwest Greenland found that ecosystem CO_2_ exchange, vegetation cover, and composition were highly sensitive to low rates (i.e., 0.5 g N m^−2^ year^−1^) of N input just 1–2 years after fertilization, suggesting that small increases in N availability have the potential to alter ecosystem structure and function in the high Arctic (Arens, Sullivan, & Welker, 2008). However, subsequent N addition from 1 to 5 g N m^−2^ year^−1^ did not further alter CO_2_ exchange or vegetation characteristics, possibly indicating ecosystem N saturation (Arens et al., 2008). In addition, recent leaf‐level work in low Arctic Alaska illuminated species‐specific decoupling of respiration and photosynthesis and shifts in leaf nutrient content across a nutrient enrichment gradient, with possible consequences for ecosystem carbon balance (Heskel, Anderson, Atkin, Turnbull, & Griffin, 2012).

In this study, we sought to examine the effects of incremental N and P enrichment on plant community composition and ecosystem function in low Arctic tundra. Specifically, we examined how plant diversity, canopy leaf area, and key components of the capacity for ecosystem C cycling (i.e., net ecosystem exchange (NEE), ecosystem respiration (ER), and gross primary productivity (GPP)) respond to a gradient of experimental N and P enrichment at a low Arctic tundra site in northern Alaska. As we were interested in how the maximum capacity for ecosystem CO_2_ exchange was impacted by the magnitude of nutrient addition, and not how nutrient addition impacts CO_2_ exchange throughout a season, we focused on measuring ecosystem processes during the period of peak tundra greenness. In addition, we explored the potential to scale up our findings from the plot to the ecosystem by comparing predictions of CO_2_ fluxes derived from a widely used Arctic ecosystem CO_2_ exchange model developed by Shaver et al. (2007) to our measured CO_2_ flux data. We also used this model to help partition CO_2_ flux responses to nutrient enrichment between various drivers (i.e., leaf area, irradiance or temperature). Overall, we hypothesized that plant diversity (e.g., species richness and abundance‐weighted diversity) and ecosystem function (e.g., NEE, GPP, ER) would respond to relatively low levels of nutrient addition.

## 2 Materials and methods

### 2.1 Site description and experimental manipulation

All field sampling for this study took place during peak growing season (i.e., the period of peak tundra greenness) across a long‐term nitrogen (N) and phosphorus (P) enrichment experiment established in 2006 by G. Shaver and colleagues at the Arctic Long Term Ecological Research (ARC LTER) site, located at Toolik Lake in the northern foothills of the Brooks Range, Alaska (68°38′N and 149°43′W, 760 m a.s.l.). The nutrient addition gradient is located on moist acidic tundra with soils comprised of 30–55 cm of a peaty organic and silty mineral layer, atop continuous permafrost. Each year, following snowmelt but before leaf‐out, granular ammonium nitrate and superphosphate is distributed on each 5 × 20 m plot, corresponding to fertilization treatment. Treatment name denotes the amount of fertilizer applied at the beginning of each growing season: “CT”, a control that receives no fertilizer; “F0.5” (0.5 g N m^−2^ year^−1^ + 0.25 g P m^−2 ^year^−1^); “F1” (1 g N m^−2^ year^−1^ + 0.5 g P m^−2 ^year^−1^); “F2” (2 g N m^−2^ year^−1^ + 1 g P m^−2^ year^−1^); “F5” (5 g N m^−2^ year^−1^ + 2.5 g P m^−2^ year^−1^); and “F10” (10 g N m^−2^ year^−1^ + 5 g P m^−2^ year^−1^). The nutrient enrichment plots are replicated in a complete three‐block design, resulting in 18 sampled treatment plots, and blocks are positioned roughly 50–100 m apart. The growing season at the ARC LTER site spans 10–12 weeks, beginning in early to mid‐June, with an average growing season temperature of 10°C. The period of peak tundra greenness for low Arctic tundra plant communities that are dominated by graminoids and evergreen shrubs is approximately 30–35 days (Sweet, Griffin, Steltzer, Gough, & Boelman, 2015). As the growing season in this system is short, we focused on measuring plant community properties and ecosystem function during the period of peak tundra greenness to ensure that we were examining the effects of nutrient addition, and not seasonality, on plant communities and the maximum capacity for ecosystem function.

To compare the magnitude of the experimental nutrient additions to that of naturally occurring fertilization, we calculated a rough estimate of thawing related nutrient enrichment. To do so, we combined data on bulk soil N from Arctic tundra soils (Mack et al., 2011), the change in annual maximum thaw depth from 2000 to 2012 at the Toolik Lake LTER (Shaver & Laundre, 2012), ANPP from Arctic tundra (Shaver, 2013), and tissue N content (Field & Mooney, 1986; Jackson, Mooney, & Schulze, 1997). Assuming steady state of the prethawing soil pool, we estimated a mineralization rate constant, which we used to estimate thawing‐driven N mineralization (see Appendix S1 for detailed calculation). According to this calculation, naturally occurring enrichment due to thawing permafrost is around 0.3 g N m^−2^ year^−1^, which falls just below the lowest nutrient enrichment treatment in our study. We suspect that the true thawing‐driven nutrient enrichment is likely lower than this (see Appendix S1).

### 2.2 Leaf area index, plant community composition, and plant diversity

To calculate leaf area index (LAI; m^2^ one‐sided green leaf per m^2^ ground), we used the Normalized Difference Vegetation Index (NDVI). Derived from reflectance data, NDVI captures the relative amount of green vegetation and thus is an indicator of canopy “greenness” (Rouse et al. 1974). NDVI has proven to be sensitive to differences in aboveground plant structure, biomass, and canopy cover in Arctic tundra ecosystems (Boelman, Gough, McLaren, & Greaves, 2011; Boelman et al., 2003; Steltzer & Welker, 2006; Vierling, Deering, & Eck, 1997). We obtained spectral reflectance measurements during peak tundra greenness (July 12–20, 2015) for a subset of at least two of our CO_2_ flux locations per plot (*N* = 39) with a field portable double channel spectrometer (UniSpec DC, PP Systems, Amsbury, MA, USA). The foreoptic was held 1 m above the top of the canopy, with a circular footprint of approximately 0.15 m^2^ and a 40 cm diameter field of view. Three measurements were made within each sampled flux quadrat (roughly 0.75 m in diameter) and averaged to capture spatial heterogeneity. Each vegetation upwelling radiance measurement was immediately followed by a measurement of a 99% reflectance standard from a white Spectralon™ disc (LabSphere, North Sutton, N.H., USA). By dividing the reflected vegetation radiance by the spectralon radiance, we obtained a value for spectral reflectance. NDVI values were calculated from spectral reflectance measurements using Equation (1), where NIR indicates reflectance at 800 nm [a near‐infrared (NIR) wavelength], and *R* is reflectance at 660 nm [a visible red (*R*) wavelength]. The NDVI values at each CO_2_ flux plot were averaged to obtain a mean value.

(1) NDVI=(NIR−R)/(NIR+R)

Mean NDVI for each flux plot was used to estimate LAI using a model developed by Street et al. (2007) for varying tundra vegetation types, generalized by Shaver et al. (2007) (Equation (2)). This model is commonly employed in studies of Arctic vegetation and carbon fluxes (Loranty et al., 2011; Shaver et al., 2013; Street et al., 2012; Sweet et al., 2015), and it assumes that differences in NDVI during the period of peak leaf‐out (when our study was conducted) are primarily the result of changes in tundra leaf area.

(2) $$\text{LAI}\;=\;0.0026e^{8.0783*\mathrm{NDVI}}$$

To examine treatment effects on plant diversity, we analyzed percent cover during the period of peak tundra greeness (July 13 – 16, 2012) using data available through the LTER data portal (http://ecosystems.mbl.edu), collected at eight 1‐m^2^ quadrats within each 5 × 20 m treatment plot). We used these percent cover data to calculate the number of species in the community, species richness (*S*), and two common abundance‐weighted diversity metrics, the Shannon Index (Equation (3)) and the Simpson Index (Equation (4)), that represent the evenness and dominance of species in a community, where *P*_*i*_ is the fraction of the community made up of species *i* and *S* is the species richness of a given community.

(3) $$H\;=\;\sum_{i=1}^{S}-\left(P_{i}*\ln P_{i}\right)$$

(4) $$D\;=\;\frac{1}{\sum_{i=1}^{S}P_{i}^{2}}$$

All measures of diversity were calculated using the vegan package (Okasen et al., 2015) in R v. 3.2.1 (R Core Team 2015). As plant percent cover data were taken at eight subplots within the control, F0.5, F2, F5, and F10 treatment plots, they are an accurate, thorough representation of plant communities across the experimental plots. Given the short growing season and the large abundance of perennial and evergreen species with conservative growth strategies in this system (Bliss & Petersen, 1992) plant communities likely shift slowly, rather than abruptly, in response to environmental change and resource availability (Camill & Clark, 2000; Dormann & Woodin, 2002). In addition, previous work in this region has shown that plant percent cover in this system is unlikely to change over short (e.g., <5 years) temporal scales (Jorgenson, Raynolds, Reynolds, & Benson, 2015).

### 2.3 Measured CO_2_ flux measurements and calculations

During the period of peak tundra greenness (July 12–16, 2015), changes in CO_2_ concentration, water vapor, photosynthetically active radiation (PAR), and air temperature were measured using a Li‐Cor 6400XT infrared gas analyzer (IRGA; Li‐Cor, Lincoln, Nebraska, USA) operated in closed‐system mode. The IRGA was affixed to a transparent, cylindrical, portable polycarbonate chamber (*r* = .36 m; *h* = 0.61 m), with internal fans to ensure adequate mixing of air and steady chamber temperatures, atop a separate base (*r* = .37 m; *h* = 0.15 m) fitted with a plastic skirt, sealed to the ground with two heavy chains. Because the range of LAI values across all plots was relatively small (Figure 4a), the same chamber was used for all gas exchange measurements. At each sampling location, we conducted flux measurements to permit calculation of both net ecosystem exchange (NEE) and ecosystem respiration (ER). Each measurement cycle began by lowering the chamber onto the base and sealing it. Once a consistent rate of CO_2_ exchange was achieved, we began logging a 40‐s flux measurement—following a method similar to the International Tundra Experiment (ITEX) and that of Shaver et al. (1998, 2007), and (Shaver et al., 2013)—in the light (for calculation of NEE) at a sampling frequency of 0.5 Hz. Once we completed a flux measurement in the light, the flux chamber was covered with an opaque black cloth and allowed to acclimate for 15–30 s before logging a 40‐s flux measurement in the dark (for calculation of ER). This cycle was repeated five times, yielding five flux measurements in the light and five in the dark at each sampling location. The temperature in the chamber did not exceed 25.2 °C during any measurement, and conditions for each repeated measure were stable. For each sampling location within each treatment plot, we averaged the five fluxes made in the light and the dark, respectively, and we calculated three relevant flux metrics: NEE, ER, and gross primary production (GPP). Measurements from the three sampling locations were averaged to obtain a mean value for each treatment plot, resulting in three observations per treatment (one mean value per treatment per block), and outliers were removed prior to averaging.

To calculate NEE (μmol m^−2^ s^−1^), we used Equation (5) to quantify the continuous exchange of CO_2_ between the atmosphere, vegetation, and soil in the light. In Equation (5), ρ is the air density (mol air per m^3^), defined as $\frac{P}{\mathrm{RT}}$, where *P* is the average pressure (Pa), *R* is the ideal gas constant (8.314 J mol^−1^ air K^−1^), and *T* (K) is the mean temperature. *V* is the chamber volume (m^3^), d*C/*d*t* is the slope of the chamber CO_2_ concentration against time (μmol CO_2_ mol^−1^ air s^−1^), and *A* is the surface area of the ground (m^2^) within the chamber. Negative NEE values indicate fluxes from the atmosphere to the ecosystem, and positive values indicate fluxes to the atmosphere from the ecosystem.

(5) NEE=(ρ∗V∗(dC/dt)/A)

(6) GPP=ER−NEE

In addition, we calculated ER using Equation (5) for all flux measurements taken in the dark. We then calculated gross primary production (GPP) as the difference between ER and NEE (Equation (6)).

### 2.4 Modeled CO_2_ fluxes

To compare our flux measurements to those predicted at a system‐level scale we modeled net ecosystem exchange (NEE_M_) using the model developed initially by Shaver et al. (2007) (Equations (7) through (9)) and further modified by Shaver et al. (2013) which requires input of three variables: LAI, PAR, and air temperature (*T*). Predicting CO_2_ fluxes using only LAI, PAR, and air *T* has been shown to produce a reasonable estimation of Arctic tundra CO_2_ exchange (Rastetter et al., 2010; Shaver et al., 2007; Street et al., 2007). While the model is often viewed as a bulk NEE model, accurate representations of ER and GPP are critical to determining realistic estimates of NEE. In addition, previous work has shown that robust estimates of NEE, particularly at the landscape scale, require an accurate and mechanistic understanding of both ER and GPP (Loranty et al., 2011).

PAR and *T* data were obtained from the Li‐Cor 6400XT used for CO_2_ flux measurements. PAR and *T* values were calculated for each of the five measurements made in the light and then averaged to obtain mean values. NEE_M_ (μmol CO_2_ m^−2^ s^−1^) was calculated using Equation (7) as the difference between modeled ER (ER_M_) and GPP (GPP_M_) where negative values of NEE_M_ represent net CO_2_ uptake by the ecosystem. While variations on the model exist, we used model parameter values estimated on low Arctic (the bioclimatic region our study was conducted in that lies between the sub‐Arctic and high Arctic) datasets that encompass a variety of low Arctic tundra vegetation types (Shaver et al., 2013).

(7) $${\text{NEE}}_{M}\;=\;{\text{ER}}_{M}-{\text{GPP}}_{M}$$

ER_M_ was calculated using Equation (8), using parameter values for β, *R*_0_, and *R*_X_ as determined by Shaver et al. (2013). Here, *R*_0_ (1.177 μmol CO_2_ m^−2^ leaf s^−1^) is the basal respiration rate, accounting for both autotrophic and heterotrophic respiration, β (0.046 per °C) is an empirically fit parameter, and *T* is air temperature (°C). The additional source of respiration in Equation (8), *R*_X_ (0.803 μmol CO_2_ m^−2^ ground s^−1^), corresponds to respiration at deeper soil horizons, is independent of LAI and fluctuations in air *T*, and is included in the model as it enhances accuracy, model fit, and prevents ER from dropping to zero when there is no canopy leaf area (Shaver et al., 2013).

(8) $${\text{ER}}_{M}\;=\;\left(R_{0}*e^{\beta *\mathrm{air}T}*\text{LAI}\right)\;+\;R_{x}$$

(9) $${\text{GPP}}_{M}\;=\;\left(P_{\max L}/k\right)*\ln\left(\left(P_{\max L}+E_{0}*I\right)/\left(P_{\max L}+E_{0}*I*e^{\left(-k*\mathrm{LAI}\right)}\right)\right)$$

Modeled gross primary productivity (GPP_M_) was calculated using Equation (9) and parameter values for *P*_maxL_, *k*, and *E*_0_ from Shaver et al. (2013), where *P*_maxL_ (14.747 μmol CO_2_ m^−2^ leaf s^−1^) is the light‐saturated photosynthetic rate per unit leaf area, *k* (0.5 m^2^ ground/m^2^ leaf) is a Beer's law extinction coefficient, and *E*_0_ (0.041 μmol CO_2_ fixed per μmol photons absorbed) is the initial slope of the light response curve. Incoming solar irradiance (*I*) is the top‐of‐the‐canopy photosynthetic photon flux density (μmol photons absorbed m^−2^ ground s^−1^). Irradiance is assumed to be the same per leaf area as per ground area at a given layer in the canopy. *I* was calculated from PAR data recorded by an upward‐looking sensor logged by the LiCor 6400XT IRGA; we calculated an average PAR value for each flux location.

### 2.5 Statistical analyses

We used a series of mixed effects models for each of our response variables (e.g., plant diversity and ecosystem function) with treatment as a fixed effect and block as a random effect. Block did not have a significant effect in any of our preliminary analyses. Therefore, to determine the influence of nutrient enrichment on measured and modeled CO_2_ fluxes, plant diversity, relative cover of plant functional groups and LAI, we used one‐way analyses of variance (ANOVA) followed by Tukey Honest Significance Difference post hoc tests when ANOVA results were significant (*N* = 3 for each treatment level). Linear models were used to compare measured and modeled flux metrics, and we characterized the strength of the relationship between measured and predicted ecosystem CO_2_ flux metrics (i.e., NEE, ER, GPP) using the coefficient of determination (*R* ^2^) and root mean squared error (RMSE). In addition, we compared a subset of our measured flux data for which we had LAI values and our modeled fluxes between nutrient treatments using a two‐way ANOVA. For all analyses, *p*‐values <.05 were considered statistically significant. All analyses were completed in R v. 3.2.1 (R Core Team 2015) using the ggplot2 (Wickham, 2009), lme4 (Bates, Maechler, Bolker, & Walker, 2015), lsmeans (Lenth, 2016), and vegan (Okasen et al., 2015) packages.

## 3 Results

### 3.1 Leaf area index, plant fractional cover, and plant diversity

**Figure 1:**
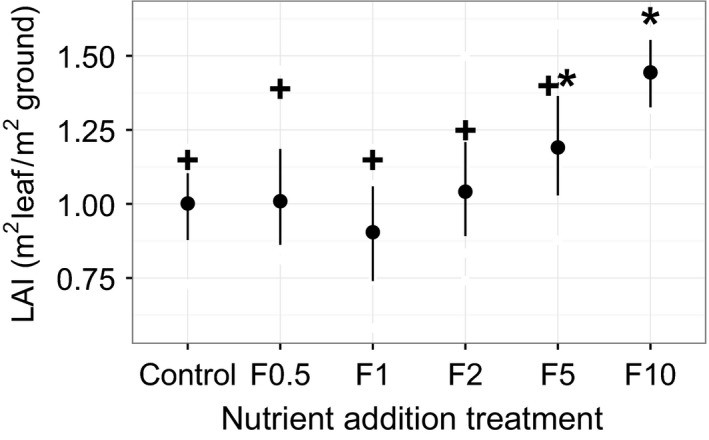
Leaf area index (LAI) across nutrient addition treatments. Points are mean LAI values (*N* = 3), and error bars represent the standard error (*SE*) of the mean. LAI increased with nutrient addition, and LAI was significantly higher at the highest treatment (F10) than in all other treatments (except for F5). Statistically significant differences are indicating by non‐overlapping symbols (i.e., * and +)

**Figure 2:**
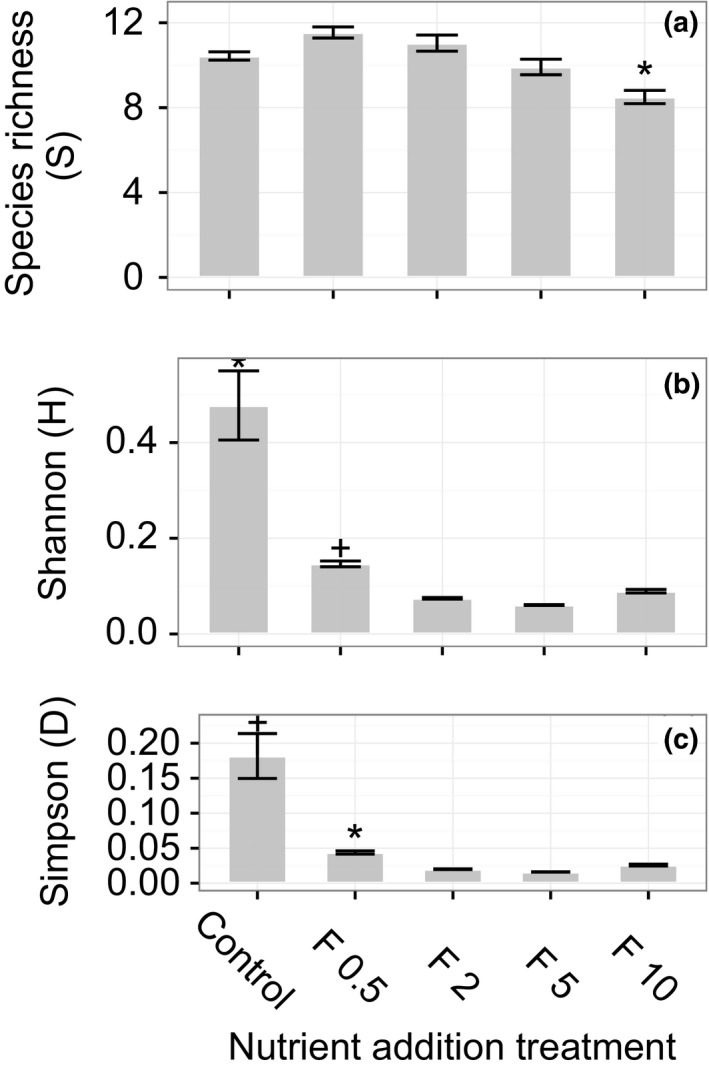
Plant diversity by nutrient addition treatment. Species richness (S) is the number of vascular plant species in a community, and the Shannon (H) and Simpson (D) values are unitless index values representing relative abundances of species in a community. Statistically significant differences are represented by nonoverlapping symbols (*N* = 3). Error bars represent the standard error (*SE*) of the mean. (a) S was significantly lower at the highest nutrient addition treatment (F10) than all other treatments and the control. There were no significant differences between the remaining treatments or between the control and addition treatments. (b, c) H and D, respectively, decreased from the control treatment to the first addition treatment (F0.5) and again between F0.5 and all other addition treatments (F2, F5 and F10)

We detected a statistically significant difference in leaf area index (LAI) with nutrient addition. LAI was significantly greater in the highest nutrient addition treatment (F10) than in all other treatment levels, except for F5 (Figure 1). Mean LAI hovered around 1 for the CT, F0.5, F1, and F2 treatments, and mean LAI was 1.19 (*SE* = 0.04) and 1.44 (*SE* = 0.09) at F5 and Fl0, respectively (Figure 1). When examining plant community composition and diversity, we found strikingly divergent trends in plant species richness, the number of species in a community (S), and two abundance‐weighted measures of plant diversity, the Shannon (H) and Simpson (D) indices, in response to nutrient addition. Species richness did not decrease significantly with nutrient addition until the highest level of enrichment (Figure 2a), when mean S dropped to 8.5 (*SE* = 0.31) compared to 10.4 (*SE* = 0.20) in control plots. However, when abundance‐weighted measures of plant diversity were considered, control plots had 69% and 76% higher H and D index values, respectively, than the lowest nutrient addition treatment (F0.5) which had 39%–59% higher H values and 41%–64% higher D values than all other treatment levels (Figure 2b,c).

In order to further examine shifts in plant communities with nutrient addition, we decomposed our diversity measures and explicitly examined changes in the percent cover of four plant functional groups: deciduous shrubs (e.g., *Betula nana*,* Salix pulchra*,* Vaccinium uligonosum*), evergreen shrubs (e.g., *Empetrum nigrum*,* Vaccinium vitis‐idea*), forbs (e.g., *Rubus* chamaemorus, *Polygonum bistorta*), and graminoid species (e.g., *Eriophorum vaginatum*,* Carex bigelowii*). We found statistically significant effects of nutrient addition on deciduous shrub, evergreen shrub and forb cover. The relative abundance of deciduous shrubs was significantly higher at F10 than in the control, and the percent cover of the dominant deciduous shrub species, *B. nana,* was higher in the F10 treatment than in CT, F0.5 and F2 treatment plots. In addition, the relative abundance of forb species was significantly higher at F10 than in control plots and at F0.5. Finally, evergreen shrub cover decreased with nutrient enrichment and was significantly lower at F10 than at F0.5 and F2, and tended toward being significantly lower at F10 than at CT and F5 (both *p *<* *.1). We did not detect statistically significant differences between treatments for graminoid (grass/sedge) cover.

### 3.2 Measured ecosystem CO_2_ fluxes

**Figure 3:**
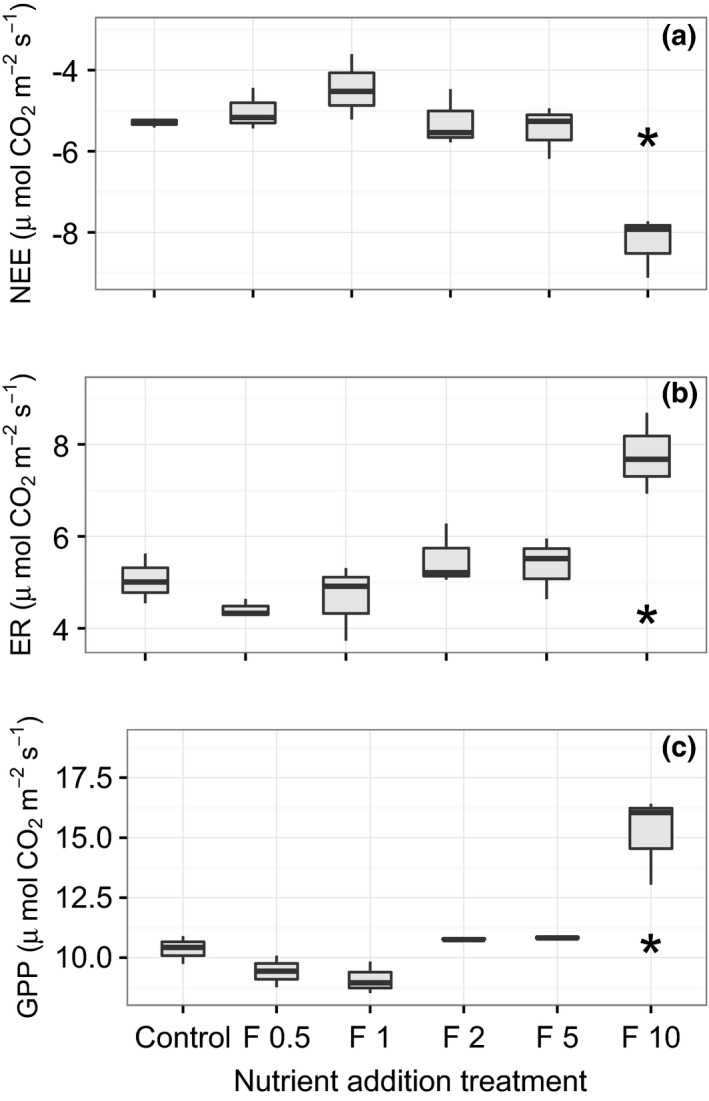
Boxplots depicting treatment differences between three measured flux variables: net ecosystem exchange (NEE), ecosystem respiration (ER), and gross primary productivity (GPP). Asterisks denote significant differences between means (*N* = 3) at the highest nutrient addition treatment (F10) for all three CO_2_ exchange metrics

Environmental conditions were relatively stable throughout the sampling period (see Figure S1 in Supporting Information), and there were no statistically significant differences in PAR or *T* across sampling dates or between nutrient addition treatments (see Figure S2 in Supporting Information). Across all fluxes and treatment plots, measured NEE ranged from −9.12 to −3.61 (*M* = −5.62, *SE* = 0.20), ER from 3.73 to 8.69 (*M* = 5.14, *SE* = 0.21), and GPP from 8.52 to 16.41 (*M* = 10.94, *SE* = 0.38), all μmol CO_2_ m^−2^ ground s^−1^. There were statistically significant differences in GPP (*p *<* *.001), NEE (*p *<* *.01) and ER (*p *<* *.05) across nutrient addition treatments. NEE values were significantly larger (NEE was more negative indicating larger fluxes to the ecosystem) in the highest nutrient addition treatment (F10) when compared to all other treatments (Figure 3a). In addition, GPP and ER were higher at F10 than at all other treatments (Figure 3b,c).

### 3.3 Measured‐modeled CO_2_ flux comparison

**Figure 4:**
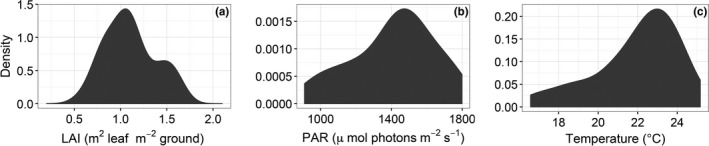
Frequency distributions of environmental data and leaf area index (LAI) collected during CO_2_ exchange measurements for the calculation of predicted CO_2_ fluxes using a widely employed Arctic ecosystem exchange model by Shaver et al. (2007). (a) LAI values ranged from 0.58 to 1.63 (*M *=* *1.11, *SE* = 0.04) m^2^ leaf/m^2^ ground. (b) Photosynthetically active radiation (PAR) ranged from 909 to 1779 (*M = *1406, *SE* = 37.07) μmol photons m^−2^ s^−1^. (c) Air temperature across all sampling locations ranged from 16.58 to 25.17 (*M = *21.94, *SE* = 0.34) °C

**Figure 5:**
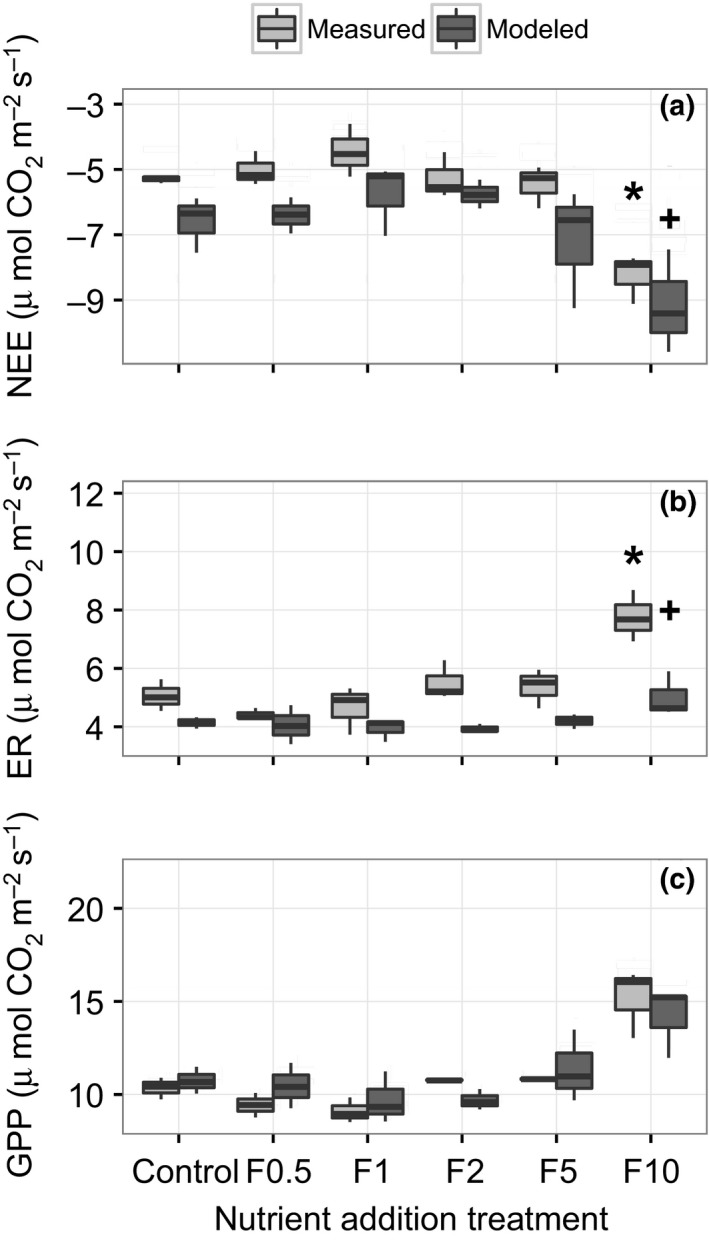
Boxplots showing a comparison of the subset of measured CO_2_ flux data (the subset for which we have leaf area index values) and modeled data across nutrient treatments (*N* = 3 for both modeled and measured data at each treatment level). Statistically significant differences between measured and modeled data within a treatment are indicated by nonoverlapping symbols (i.e., * and +). Measured ER values were significantly higher than modeled values in the F10 treatment. There were no statistically significant differences between measured and modeled NEE or GPP by nonoverlapping symbols (i.e., * and +)

We calculated modeled NEE, GPP, and ER using a model that required the input of three measured variables: LAI, PAR, and *T*. LAI ranged from 0.58 to 1.63 (*M *=* *1.11, *SE* = 0.04) m^2^ leaf/m^2^ ground, PAR ranged from 909 to 1,779 (*M = *1406, *SE* = 37.07) μmol photons m^−2 ^ground s^−1^, and *T* from 16.58 to 25.17 (*M = *21.94, *SE* = 0.34) °C (Figure 4a–c). For modeled values, NEE_M_ ranged from −11.20 to −3.41 (*M *=* *−7.09, *SE* = 0.30), ER_M_ from 2.47 to 6.28 (*M *=* *4.38, *SE* = 0.14), and GPP_M_ from 5.88 to 16.28 (*M *=* *11.47, *SE* = 0.42), all μmol CO_2_ m^−2^ ground s^−1^. We found similar trends for modeled NEE and GPP as those observed for measured fluxes. NEE and GPP were significantly greater (more negative in the case of NEE) at F10 than all other treatments except for F5 (Figure 5a,c). We did not find any significant differences in modeled ER across nutrient addition treatments (Figure 5b).

**Figure 6:**
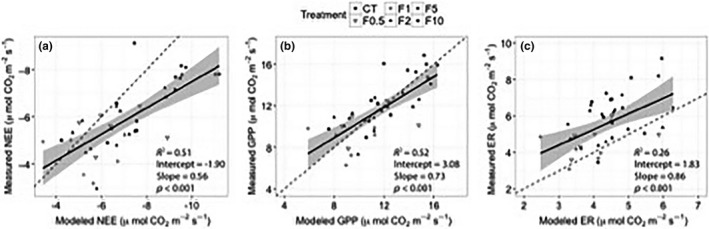
Relationships between measured and modeled CO_2_ flux variables. Predicted fluxes were calculated using a widely employed Arctic CO_2_ exchange model by Shaver et al. (2007). All plots include a solid linear regression line, a shaded 95% confidence interval of the regression line, and a dashed one to one line. (a) The model explained 51% of the variance in net ecosystem exchange (NEE); (b) 52% of the variance in gross primary production (GPP); and (c) it explained 26% of the variance in ecosystem respiration (ER)

When we compared our measured flux data to the model developed by Shaver et al. (2007), we found that the model explained 50.9% of the variance in NEE in our dataset, and the root mean square error (RMSE) for measured versus modeled NEE was 1.29 μmol CO_2_ m^−2^ ground s^−1^ (Figure 6a). For GPP, the regression explained 52.4% of the variance and the RMSE was 1.76 μmol CO_2_ m^−2^ ground s^−1^ (Figure 6b). The model explained less of the variance for ER (25.9%) with a RMSE of 1.71 μmol CO_2_ m^−2^ ground s^−1^ (Figure 6c). To assess the role of LAI in our modeled flux calculations, as opposed to temperature or PAR, we re‐calculated our modeled fluxes using randomized LAI values across our dataset, and we found that the model explained less than 8.5% of the variance for all flux variables (i.e., NEE, ER, GPP), with no significant slopes (all *p *>* *.05). The relationship between modeled and measured data appeared to weaken at the highest level of nutrient addition. To examine potential differences between measured and modeled fluxes at the highest nutrient addition treatment, we compared the subset of our measured data for which we had LAI data (Figure 5a–c) to the modeled CO_2_ flux data by treatment level. We found significant differences between measured ER and modeled ER with nutrient addition, and measured ER was significantly higher than modeled ER at the F10 treatment (Figure 5b). We did not detect statistically significant differences between measured and modeled NEE or GPP with nutrient addition.

## 4 Discussion

The goals of this study were to assess how incremental nutrient additions ranging from small to large doses impacted Arctic tundra (1) plant community properties and (2) key components of the capacity for ecosystem carbon cycling during the period of peak tundra greenness. Warming‐induced nutrient enrichment is presumably a gradual process, and understanding the responses of plant communities and ecosystem function to relatively low levels of nutrient fertilization is a crucial step in predicting ecological responses to global change. We found that a gradient of nutrient enrichment revealed nonlinear responses of plant communities and the capacity for ecosystem CO_2_ exchange to nutrient manipulations at a low Arctic site, largely deviating from our initial hypotheses. This study advances our understanding of the responses of Arctic plant communities and the capacity for ecosystem function to scenarios of gradual nutrient enrichment that are likely to be more indicative of warming‐induced shifts in nutrient availability than additions of large amounts of N and P that were designed to illustrate and understand Arctic tundra nutrient limitation.

### 4.1 Plant diversity declines with small increases in nutrient availability

Consistent with our hypotheses, we found that plant diversity indices that account for species evenness and dominance declined with just a small amount of nutrient addition (Figure 2b,c)—the level that is most comparable to our estimate of thawing‐induced enrichment. However, nutrient enrichment did not affect species richness (*S*) until high levels of addition (Figure 2a). Nutrient‐limited ecosystems are often characterized by plant communities with species that differ strongly in their ability to respond to alterations in resource availability; thus, shifts in plant diversity or in species‐specific, leaf‐level physiology may precede any changes in ecosystem processes and properties (Aber et al., 1998). Long‐term (i.e., >20 years) Arctic tundra enrichment experiments with additions comparable to the highest (F10) treatment in this study have documented declines in species richness and shifts in species dominance and evenness with nutrient enrichment (Gough & Hobbie, 2003; Gough, Wookey, & Shaver, 2002), as have studies in other biomes (Suding et al., 2005; Zavaleta et al., 2003). However, our findings indicate that after 6 years of enrichment, plant diversity measures that capture relative abundance are nearly as sensitive to low levels of nutrient fertilization as they are to high levels.

There are a variety of ecological mechanisms that might explain shifts in abundance‐weighted plant diversity, but not species richness, with low‐to‐moderate levels of nutrient addition. Lower levels of nutrient enrichment of a N‐ and P‐limited system may reduce niche differentiation or complementarity that would otherwise promote species coexistence (Harpole et al., 2011), leading to increases in the relative abundance of species that outcompete neighbors with lower growth rates (Hautier, Niklaus, & Hector, 2009). We found that large increases in nutrient availability (i.e., F10) ultimately favored deciduous shrub and forb species and led to declines in evergreen shrub cover. These findings are in keeping with previous work in this system that found that after 6 years of N and P fertilization comparable to the F10 treatment in our study, increases in LAI of the dominant deciduous shrub species, *Betula nana*, and the formation of a dense canopy resulted in light limitation of other shrub species and plant functional types (Bret‐Harte et al., 2001). In addition, previous work across our study gradient found that *B. nana* foliar N increased at high levels of fertilization (e.g., F10), but not at low levels, and found no effect of fertilization on foliar N of the dominant graminoid species, *Eriophorum vaginatum* (Heskel et al., 2012). Here, we found that LAI increased with nutrient addition (Figure 1), as species evenness declined, ultimately resulting in changes in ecosystem CO_2_ exchange at high levels of nutrient addition, likely due to the competitive advantage of deciduous shrub species.

### 4.2 High levels of nutrient enrichment impact ecosystem CO_2_ exchange

Although low levels of nutrient addition led to declines in plant diversity via shifts in species evenness and dominance, the capacity for ecosystem CO_2_ exchange was not impacted until high levels of addition. We found that 9 years of nutrient enrichment had significant effects on NEE, ER and GPP at the highest level of addition when NEE became significantly more negative (greater fluxes to the ecosystem) because increasing GPP overcame increasing ER (Figure 4a–c). The documented responses of plant communities and ecosystem functioning to gradual addition may be due to a variety of mechanisms; however, we focus on three primary explanations: (1) the ecosystem‐level consequences of plant adaptations and responses to chronic nutrient limitation, (2) the role of microbial activity in mediating ecosystem function, and (3) abiotic nutrient sinks and/or losses.

First, as the availability and subsequent uptake of limiting resources is predicted to stimulate primary productivity, we expected to see an immediate increase in GPP and NEE after 9 years of low or moderate nutrient enrichment; however, plant communities across chronically nutrient‐limited landscapes may exhibit lower overall maximum potential growth rates or may allocate resources to belowground structures in order to maximize nutrient uptake and retention (Chapin, 1980, 1991; Chapin, Vitousek, & Vancleve, 1986; Grime, 1977). Thus, increasing nutrient availability may not be diverted to the production of photosynthetic biomass, inducing shifts in below versus aboveground allocation with functional consequences for ecosystem CO_2_ exchange, until high levels of fertilization.

Second, nitrogen (N) and phosphorus (P) mineralization in Arctic soils is thought to be low during the growing season, linked to the immobilization of nutrients by microorganisms, contrasted with a high release rate during the winter (Giblin, Nadelhoffer, Shaver, Laundre, & Mckerrow, 1991; Nadelhoffer, Giblin, Shaver, & Laundre, 1991). These findings point to competition between plants and microbes during the growing season that might explain the lag in GPP until a high level of enrichment is reached, possibly driving the decoupling of plant community and ecosystem responses to multilevel nutrient enrichment. While Arctic tundra plant communities are known to be nutrient‐limited, tundra microbial communities are also nutrient‐limited, as is evidenced by the stimulation of microbial N‐immobilization and enhancement of microbial activity with nutrient enrichment (Lavoie, Mack, & Schuur, 2011; Mack et al., 2004). As a result, nutrient enrichment effects on ecosystem‐level process, and specifically the stimulation of ecosystem CO_2_ exchange, may not be seen until high levels of fertilization when nutrient availability outpaces microbial utilization.

Finally, there are two abiotic mechanisms that may be responsible for our documented ecosystem‐level responses to a gradient of nutrient enrichment: abiotic sinks and leaching. First, abiotic sinks via the adsorption and precipitation of P might initially compete with plants and microorganisms for increasing P availability (Olander & Vitousek, 2004), ultimately resulting in a P sink that is not saturated until high levels of experimental P addition (i.e., the F10 treatment in this study). While strong abiotic P sinks are well known, abiotic sinks for N are less well understood, but possibly play a significant role. In addition, leaching of dissolved organic N and nitrate and denitrification may be important loss pathways in this system (Giblin et al., 1991; Mack et al., 2004). Abiotic mechanisms may have dampened the effects of lower levels of nutrient addition, but it is not clear how they might help explain the contrasting responses of plant communities to low levels of addition and ecosystem responses to high levels of enrichment.

### 4.3 Modeled CO_2_ fluxes estimate ecosystem responses to low‐to‐moderate levels of fertilization

As the Arctic continues to warm, our ability to accurately measure, monitor and predict C cycling across large spatial and temporal scales is paramount. This task is challenging as Arctic tundra landscapes are complex and heterogeneous, and are often dominated by varying plant functional groups, with important effects on key components of C cycling (Chapin et al., 2006). However, previous research has shown that canopy C exchange across a wide range of Arctic ecosystems is controlled by the same factors despite pronounced differences in plant community composition, providing evidence of functional convergence (Street et al., 2007; Williams & Rastetter, 1999; Williams et al., 2001). As such, modeling efforts have assumed that, regardless of plant diversity or community structure, canopy C exchange can be predicted from leaf area, light and temperature alone (Shaver et al., 2007). Less is known, however, about how canopy C exchange is impacted by increasing nutrient availability.

When comparing our measured fluxes with fluxes calculated from an Arctic ecosystem exchange model by Shaver et al. (2007), we found that the model explained less of the variance than found in previous studies (e.g., Shaver et al., 2013), although it still explained roughly half of the variance for both NEE (51%) and GPP (52%), and 23% of the variance in ER (Figure 6a–c). The amount of variance explained dropped to <8.5% for all three flux variables when we randomized LAI values in our dataset, suggesting that the impact of nutrient addition on LAI, and not temperature or irradiance, is the principle driver of the variation we can account for with this model. However, given the relatively low amount of variance explained for ER, nutrient enrichment appears to have an effect on ER that cannot be explained by canopy leaf area. This effect is perhaps driven by the response of microbial communities to fertilization that is not captured by the parameter in the Shaver et al. (2007) model that represents microbial respiration from deeper soil horizons (*R*_X_), although it is surprising that this is not significant until the highest nutrient addition treatment (Figure 5b). As previous work in this system has shown that nutrient enrichment stimulates the decomposition of C pools in deeper soil horizons (Mack et al., 2004), incorporating variable *R*_X_ values into the model may help account for this discrepancy.

The discrepancy between our measured and modeled data under high nutrient fertilization may also be explained in part by the effects of background reflectance (e.g., the effects of soil or nonfoliar vegetation reflectance) on the relationship between NDVI and LAI (Rocha & Shaver, 2011). The potential impact of background reflectance, coupled with our observed shifts in community composition and possibly canopy architecture, suggest that NDVI derived LAI may not be an appropriate leaf area estimate for structurally diverse canopies. In addition, Shaver et al. (2007, 2013) suggest that the success of the model in predicting NEE using just LAI or whole canopy N content is due to a high degree of convergence in canopy structure and function, and our results suggest that high levels of nutrient addition may alter this relationship.

Overall, our data comparison demonstrates that the Shaver et al. (2007) model estimates NEE and GPP relatively well even when plant communities are subjected to resource manipulations (Shaver et al., 2013). However, the diminished ability of the model to accurately estimate ER (Figure 5b), particularly at high levels of nutrient addition, suggests that further work is needed to understand how to model ecosystem responses to nutrient enrichment. As Arctic systems continue to warm rapidly, accurate estimates of ecosystem CO_2_ exchange will be a crucial component of understanding and predicting responses and feedbacks to global change, and our findings suggest that increasing nutrient availability may impact our ability to rely on current model parameterizations.

### 4.4 Implications

To date, results from long‐term experiments examining the impacts of large annual doses of nutrients in Arctic tundra have documented significant shifts in plant community composition and dominance, aboveground biomass, and ecosystem function. However, this level of fertilization may be an unrealistic outcome of warming‐induced nutrient enrichment in the Arctic. Our study is one of the first to examine how Arctic plant communities and the capacity for ecosystem function (e.g., CO_2_ exchange) during the period of peak tundra greenness respond to a gradient of enrichment. We demonstrate that, despite reorganization of plant communities with low levels of addition, significant alteration of ecosystem CO_2_ exchange only occurs at the highest level of nutrient enrichment, suggesting a shift in the capacity for ecosystem C gain only at high levels of fertilization that likely exceed warming‐induced enrichment. In addition, we show that examining a gradient of nutrient addition may help identify thresholds past which models intended to upscale estimates of ecosystem function may decline in accuracy—improving our ability to model and comprehend the impacts of global change. In addition, our results point to the need for further work examining the role of the magnitude of nutrient enrichment on below and aboveground plant community properties and ecosystem processes and any temporal variation in these patterns and relationships.

## Conflict of Interest

None declared.

## Author Contributions

CMP and KLG conceived the ideas and designed methodology; CMP, HEG, and KLG collected the data; CMP analyzed the data; CMP and DNLM calculated/estimated naturally occurring nutrient enrichment; all authors contributed to the interpretation of analyses; CMP led the writing of the manuscript; all authors contributed critically to the drafts and gave final approval for publication.
