# Corrigendum

**DOI:** 10.1002/ece3.3079

**Published:** 2017-06-12

Prager CM. Corrigendum to “A gradient of nutrient enrichment reveals nonlinear impacts of fertilization on Arctic plant diversity and ecosystem function”. *Ecol Evol*. 2017;7:4072. https://doi.org/10.1002/ece3.3079

In a recent paper (Prager et al., 2007), we presented results for abundance‐weighted plant diversity measures generated by a plant community composition data file that miscoded plot ID as species abundance. We recalculated the Shannon and Simpson indices and find that they now follow a similar nonlinear trend as species richness and ecosystem function. These changes are reflected in an updated Figure 2 (a–c).

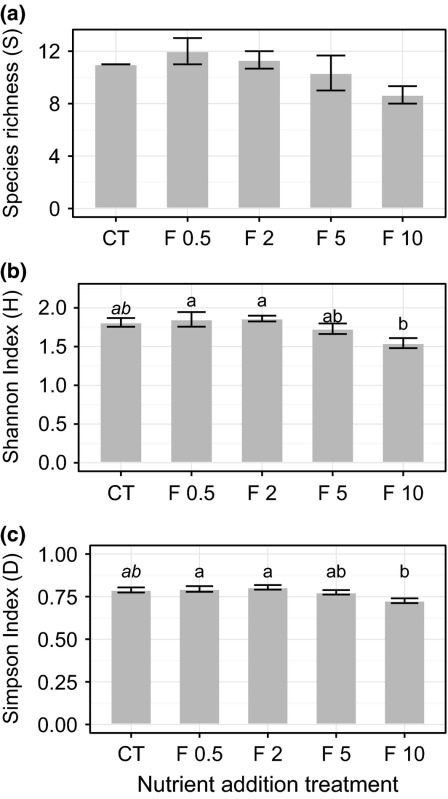

The abstract should reflect this change as “Our results suggest that only relatively large levels of fertilization … significantly alter the capacity for plant diversity and CO_2_ exchange in Arctic tundra.” In the discussion, the plant diversity subheading should now read “*Plant diversity declines with increasing nutrient availability”* followed by “We found that nutrient enrichment did not affect species richness (*S*) or abundance‐weighted diversity indices until high levels of addition (Fig. 2a–c).”

We thank Laura Gough for uncovering our error, and we also acknowledge additional funding sources (NSF 1026843 and 1637459). We provide additional information about the plant composition data used for our plant diversity calculations below (Gough, 2015).

## AUTHOR CONTRIBUTION

CMP corrected the article, all authors approved the correction.
