# Supplementary figures and images for: A gradient of nutrient enrichment reveals nonlinear impacts of fertilization on Arctic plant diversity and ecosystem function
Source: Ecol Evol. 2017 Mar 22;7(7):2449–60. doi: 10.1002/ece3.2863 (PMC5383475; doi:10.1002/ece3.2863)

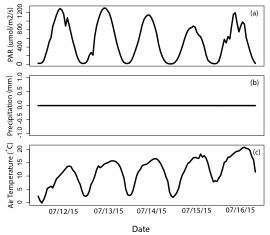

Supplement: Supplementary file 1 [file ECE3-7-2449-s001.jpg]

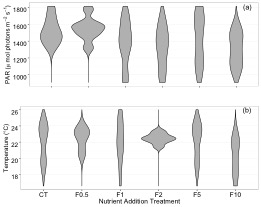

Supplement: Supplementary file 2 [file ECE3-7-2449-s002.jpg]
