# Supplementary material for: A gradient of nutrient enrichment reveals nonlinear impacts of fertilization on Arctic plant diversity and ecosystem function
Source: Ecol Evol. 2017 Mar 22;7(7):2449–60. doi: 10.1002/ece3.2863 (PMC5383475; doi:10.1002/ece3.2863)
Supplement: Supplementary file 3 [file ECE3-7-2449-s003.docx]

# Supplemental Material - S1

#################################################################

#Appendix S1. R Code used to calculate an estimate of naturally #occurring #enrichment based on existing, published data on bulk #soil N from Arctic tundra soils, the change in annual maximum #thaw depth across 12 years at the Toolik Lake LTER, ANPP from #Arctic tundra, and tissue N content estimates. In this #calculation we assume steady state of the pre-thawing soil pool, #and we estimated a mineralization rate constant, which we used #to calculate thawing-driven N #mineralization. We estimate #naturally occurring enrichment due to thawing permafrost at #around 0.3 g N m^-2^ yr^-1^, and we note that true thawing-driven #nutrient enrichment is likely lower than this estimate.

#################################################################

# dN/dt = I + P - kN

# Calculating changes in plant available N with increasing active layer depth in order to qualitatively compare the experimental treatments used in our study to an estimate of naturally occuring enrichment.

# Where,

# N is total N in soil (stratification by depth)

# I is thawing input

# P is the N coming into the soil from plant turnover

# k is mineralization constant, presumed to be equal across soil depths

# The assumption that the mineralization constant is equal across soil depths intentionally errs on the high side. In reality, the mineralization rate at the depth of thawing is likely to be substantially lower than the average across soil depth. Therefore, thawing-driven mineralization is likely lower than this estimate suggests.

# From Mack et al. (2011), we calculated a total "starting" N stock, Nhat, which we also assume is the pre-thawing equilibrium.

Nhat <- 620 # g N/m2

# From Mack et al. (2011), we took the average total N (g N/m2) at a depth of 30-35 cm (the deepest layer likely to most closely resemble that in thawed soils) at an unburned site (site 10) used in the study, and we then calculated the thawing input, I, to the organic N pool by multiplying average N (g N/m2) by the thaw rate (1.4 cm/yr) calculated based on late season thaw depth measurements in control plots at the Toolik LTER long-term moist acidic tundra fertilization experiment from 2000 to 2012 (Shaver & Laundre, 2012).

I <- 40 # g N/m2

# We also estimated the N input from NPP (P). Here, we use a tundra NPP value from Shaver (2013), a published dataset from 1982 at Toolik Lake, AK that estimates NPP (above and belowground) at 430 g biomass /m2. We assume that the same amount of biomass is returned to the soil annually as is created by NPP, and that the N content of biomass returned to the soil is 1%, which is near the low end of averages for foliar and root N (Field & Mooney, 1986, Jackson et al., 1997).

P <- 4.3 # g N/m2

# We calculated the mineralization rate, k, assuming a steady state of the pre-thawing pool (so I = 0), and no changes in rate by depth.

# Algebraically, from dN/dt = 0, k = P/Nhat

k <- P/Nhat

# This is run over time. Solving dN/dt = I + P - kN gives N(t) = (I+P)/k + (Nhat - (I+P)/k)*exp(-k*t)

t <- 0:10 # yr

Nt <- (I+P)/k + (Nhat - (I+P)/k)*exp(-k*t) # g N/m2

# Mineralized N at time t is k*Nt

Nmin <- k*Nt # g N/m2/yr

# We then calculate the annual increase in N mineralization as,

Nmin_anninc <- Nmin[2:length(Nmin)]-Nmin[1:(length(Nmin)-1)]
